# Strain-level variation controls nutrient niche occupancy by health-associated *Anaerostipes hadrus*

**DOI:** 10.1093/ismeco/ycaf163

**Published:** 2025-09-17

**Authors:** Loudon Herold, Bradley G Fitzgerald, Gwenno M E Leclercq, Matthew T Sorbara

**Affiliations:** Department of Molecular and Cellular Biology, University of Guelph, Guelph, ON, N1G 2W1, Canada; Department of Molecular and Cellular Biology, University of Guelph, Guelph, ON, N1G 2W1, Canada; Department of Molecular and Cellular Biology, University of Guelph, Guelph, ON, N1G 2W1, Canada; Department of Molecular and Cellular Biology, University of Guelph, Guelph, ON, N1G 2W1, Canada

**Keywords:** carbohydrate utilization, nutrient niche, strain-level genomic and function variation, butyrate, propionate, short-chain fatty acids, Lachnospiraceae, Anaerostipes, interaction between strains

## Abstract

Nutrient niche access by the gut microbiota impacts community assembly and dynamics, the production of host-benefiting short-chain fatty acids (SCFAs), and pathogen inhibition through colonization resistance. Furthermore, deciphering if and how niche access varies on a strain level will be important as individual strains of gut microbes are selected for inclusion in new live biotherapeutic products. Despite this, for many gut anaerobes, nutrient niche occupancy and impacts of strain variation remain unknown. Here, we examined nutrient niches of *Anaerostipes hadrus* (AH)*,* a butyrate-producing member of the *Lachnospiraceae* family. We found that AH isolates encode a carbohydrate metabolism gene repertoire that is distinct from other *Lachnospiraceae*. Furthermore, tested AH isolates show variation in carbohydrate-related genes between strains and large numbers of genes associated with horizontal gene transfer events. Functionally, we demonstrate that AH isolates exhibit strain-specific patterns of nutrient niche access that can be associated with the gain, loss, and disruption of gene clusters enabling specific carbohydrate metabolism. This strain-specific carbohydrate use drives variable SCFA production. Unexpectedly, strains exhibit differential preferences for carbohydrates, which alter SCFA profiles in environments with multiple possible nutrient niches available. Furthermore, when strains of AH interact in an environment with multiple nutrient niches available, strain–strain interactions result in varying SCFA profiles that extend beyond the additive effects of individual strain behavior. Altogether, these results demonstrate the importance of evaluating strain-level variation in the design of future live biotherapeutic products.

## Introduction

Short-chain fatty acids (SCFAs), including acetate, propionate, and butyrate, are produced through carbohydrate fermentation by the gut microbiota and have host-benefiting functions [1]. Carbohydrates include simple sugars, referring to monosaccharides and disaccharides, and complex carbohydrates, referring to oligosaccharides and polysaccharides [2]. While dietary simple sugars are highly absorbed in the small intestine, some reach the large intestine to be metabolized by the colonic microbiota [3]. In contrast, many complex carbohydrates are not readily absorbed in the small intestine and are fermented by the colonic microbiota [4, 5]. Changes in diet and microbiota-accessible carbohydrates alter community composition and host interactions [6]. Therefore, understanding how the microbiota uses carbohydrates is critical to elucidating impacts on host health. Despite this, carbohydrate use by many members of the microbiota remains poorly characterized.

While environmental parameters and stressors are also important, nutrient availability plays a major role in defining ecological niches [7, 8]. The nutrient niche theory states that a microbe must occupy a distinct ecological niche by competing for a limiting nutrient [7]. Therefore, the composition of the gut microbiota reflects how efficiently members of the community are able to use different available nutrients, including carbohydrates [7, 9]. In agreement, in defined model communities, changes in community composition can be explained by changes in specific dietary components [10–12]. In addition, nutrient niche occupancy and competition for resources play a critical role in colonization resistance [13], and are essential for inhibition of *Escherichia coli* and *Klebsiella pneumoniae* expansion [13–16].

To metabolize simple carbohydrates, bacteria employ membrane transport systems for sugar uptake, including phosphotransferase systems (PTS) and adenosine triphosphate-(ATP) binding cassette (ABC) transporters [17, 18]. To use larger complex polysaccharides, microbes typically require multiple proteins for cleaving, transporting, and metabolizing substrates. These proteins are often organized into polysaccharide utilization loci (PUL) [19, 20]. Enzymes that act on carbohydrates are termed carbohydrate-active enzymes (CAZymes), and include glycoside hydrolase (GH) protein families that cleave specific bonds between sugars [2, 21, 22]. Carbohydrate use and PULs have been best studied in the Bacteroidota phylum and remain less well characterized within the Bacillota phylum, including members of the *Lachnospiraceae* family [9, 19].

*Lachnospiraceae* are a Gram-positive family of anaerobes that produce SCFA including butyrate [23]. Notably, *Lachnospiraceae* can be members of defined consortia that provide colonization resistance against *Enterobacteriaceae* such as *Klebsiella* or *Salmonella*, or *Clostridioides difficile* [24–27], and can contribute to colonization resistance by competing for nutrient niches and producing pathogen-inhibiting SCFAs [26]. In addition, butyrate production by the microbiota is essential for regulating immune responses and maintaining cell barrier function [28–32]. In agreement, lactulose use and resulting butyrate production by a member of *Anaerostipes*, a genus within *Lachnospiraceae*, reduces food allergy in mice [33]. However, overall, little is known about nutrient niche occupancy by *Lachnospiraceae*. Furthermore, *Lachnospiraceae* show extensive strain-level genomic diversity, including in genes for carbohydrate use [23], but the functional impact of this diversity on nutrient niche occupancy remains unclear.

Here, we examined carbohydrate utilization by 19 strains of AH, a butyrate-producing *Lachnospiraceae* [23, 34]. We identified extensive strain-level variation in carbohydrate use indicating a varying capacity to occupy niche space. This is linked to strain-level genomic variation including specific carbohydrate use gene clusters. Variation in carbohydrate use or preference and complex interactions between strains alter SCFA production by individual or co-cultured AH isolates. Overall, these findings may aid the development of future therapeutics that target the restoration of AH and butyrate production [35].

## Materials and methods

*Strains:* 24 AH and *Blautia wexlerae* (BW) isolates were used [23] (Strain details: Table S1). Inoculums for all assays were prepared from streak plates on Brain Heart Infusion agar supplemented with 0.1% w/v L-cysteine and 5 g/L yeast extract. All experiments were conducted in a Coy anaerobic chamber (Coy Laboratory Products) supplied with 90% N_2_, 5% H_2_, 5% CO_2_ and equipped with a dehumidifier system. Assays were performed in 96-well plates.

*Isolate biobank analyses:* Genomes of 3023 isolates from published biobanks [23, 36, 37] were annotated via Prokka and dbCAN3 using HMMER [38, 39] and the number of CAZymes and PTS and ABC transporters was determined. CAZymes counts were based on unique family/subfamily, and transporter counts were based on annotation as a unique transporter system. A uniform manifold approximation and projection (UMAP) [40] plot was generated based on GH annotation and carbohydrate transporter annotation using Manhattan distance with the nearest neighbor and minimum distance set to 100 and 0.1, respectively.

*AH Strain Bioinformatic Analyses:* Phylogenetic relationships were evaluated based on pairwise comparisons of 16S rRNA similarity, and average nucleotide identity (ANI) via fastANI [41].

To identify carbohydrate related genes, several annotation pipelines were integrated to include annotation of PTS and ABC transporters by Prokka, GH and CAZymes with dbCAN, and KEGG annotations of carbohydrate metabolism genes [38, 39, 42]. Anvi’o was used to visualize differences in carbohydrate-related gene repertoires between isolates based on their unique GH families and subfamilies, transporter genes, and carbohydrate metabolism genes [43].

Genes from AH isolates were analyzed with HGTector2 for putative horizontal gene transfer (HGT)-derived gene assignments using default settings [44] and the proportions of glycoside hydrolase (GHs), carbohydrate transporters, and other carbohydrate metabolism genes predicted to be HGT-derived or non-HGT were compared. Mobile genetic elements (MGEs) were identified using geNomad [45] with default settings.

*Carbon source screening*: Biolog PM1 and PM2 plates were used to screen carbon source use (Biolog, Inc.) (see Table S2). IF-0a GN/GP base inoculating fluid was prepared according to manufacturer’s instructions. Colonies were first suspended in 5 ml of the prepared inoculating fluid. From this suspension, 750 μl was transferred into 9.1 ml of inoculating fluid with 2.1 ml H_2_O, and 40 μl 20% w/v yeast extract. 100 μl/well was added to the PM1/2 plates. Within the anaerobic chamber, plates were incubated in a BioTek Epoch 2 (Agilent Technologies) (37°C, OD_750_ measurement, 24 h). Two independent runs per plate per isolate were performed. The area under the curve (AUC) was calculated using the “growthcurver” R package [46]. A heatmap was plotted showing the mean value, clustering isolates with a Euclidean distance metric.

*Correlating functional and genomic variation*. For functional variation: clustering used the carbon source AUC heatmap. For genomic variation: first, variation in Prokka and dbCAN annotations was assessed based on the presence:absence and number of each annotated gene with isolates clustered by Euclidean distance. Second, this general annotation was filtered to include only PTS and ABC transporters, GHs, and carbohydrate metabolism genes annotated via Prokka, dbCAN, and KEGG, respectively. Third, a single-copy gene set-based phylogeny was generated with GtoTree using the *Bacillota* phylum as the core genome profile [47]. Tanglegrams were generated comparing functional variation with the three genomic variation measures and a cophenetic correlation coefficient was calculated using the “dendextend” R package [48].

*Gene clusters:* Comparative genomic analysis using Prokka and dbCAN annotations was used to identify gene clusters for trehalose, inositol, lactose, and stachyose metabolism. Genes flanking a given gene cluster from a representative isolate were used as a query to perform a local nucleotide BLAST against all other genes present in the same genomic region for all isolates to identify conserved flanking regions [49].

An interrupted lactose carbohydrate gene cluster was annotated in AH11, AH12, and AH13. DNA was extracted from AH11 for long-read sequencing. DNA extraction was carried out using the QIAGEN QIAamp PowerFecal Pro DNA kit. Samples were submitted to Plasmidsaurus, Inc. for long-read sequencing prior to annotation as described above.

*Growth for SCFA measurement:* AH strains were suspended in prereduced phosphate buffered saline (PBS) and inoculated in 50% BHI without dextrose (BHI-D Alpha Biosciences, MD) supplemented with 5% or 0.5% w/v dextrose, inositol, lactulose, lactose, or trehalose as indicated. For conditions with two carbohydrates, 2.5%/0.25% inositol and either 2.5%/0.25% lactose or trehalose were added for a total of 5% or 0.5% w/v. Culture supernatants were collected at the indicated timepoints from 0–24 h and stored at −80°C. For growth curves, continuous OD_600_ measurements were recorded for isolates grown in BHI-D with 5% w/v inositol or 5% w/v lactose supplementation.

SCFAs were measured using a pentafluorobenzyl bromide (PFB-Br) derivatization method [50]. 100 μl of supernatant and 400 μl of methanol containing D3-acetate and D7-butyrate internal standards was mixed and centrifuged (13 000 g, 4°C, 18 min). 100 μl was added to a glass vial with 100 μl borate buffer, 400 μl acetonitrile with 150 mM PFB-Br, and 600 μl hexane and incubated (65°C, 1 h, 1500 rpm). 400 μl of the organic layer was transferred to a vial and 1 μl was injected with an Agilent GC-MS (5977B) with a 10:1 split using negative chemical ionization mode with helium (carrier gas) and methane (reagent gas). SCFA peaks were identified using Mass Hunter Quantitative and normalized to internal standards and quantified using a standard curve.

Carbohydrates were measured using trimethylsilylation derivatization. A mixture of 100 μl culture supernatant and 400 μl methanol containing D6-succinic acid internal standard was centrifuged (13 000 g, 4°C, 18 min) prior to storage (−80°C, 24 h). 100 μl was added to a glass vial and dried (15 l/min nitrogen, 30 min, 25°C). Dried samples were resuspended in 50 μl of 20 mg/ml methoxyamine in pyridine and incubated (30°C, 90 min, 1400 rpm). Next, 160 μl of BSTFA with 1% TMCS (Sigma Aldrich) and 140 μl of ethyl acetate was added and samples were incubated (70°C, 60 min, 1500 rpm). 400 μl of ethyl acetate was added and 1 μl was injected with an Agilent GC-MS (5975C) with a 20:1 split using electron impact mode. Carbohydrate peaks were identified using Mass Hunter Quantitative and normalized to internal standards.

Quantitative polymerase chain reaction (qPCR): AH co-cultures were pelleted and DNA was extracted with the Quick-DNA Fecal/Soil Microbe Microprep Kit (Zymo) and diluted 1:20 in water. Reactions were prepared with 10 μl qPCR mix (Power SYBR Green), 1 μl each forward/reverse primers (Table S3), 2 μl water, and 6 μl of DNA. Plates were sealed and centrifuged (500 g, 30 s). qPCR was performed on a BIORAD iQ5 following manufacturer protocols. DNA concentration was determined by quantification using a standard curve.

### Statistical analysis

Statistical analyses were performed in R studio. A two-tailed t-test was used to compare the proportions of HGT-derived or non-HGT genes, and butyrate and propionate levels or AH strain abundance in co-cultures (Figs 2B,8F and G). ^*^,^**^,^***^,^****^, indicate *P* < .05, .01, .001, .0001, respectively. All other analyses used a one-way ANOVA followed by a *post hoc* Tukey test, with letters indicating statistical significance. *P*-values <.05 were considered significant. All raw data are provided in Table S4.

## Results

### AH strains exhibit diversity in genes for carbohydrate use

**Figure 1:**
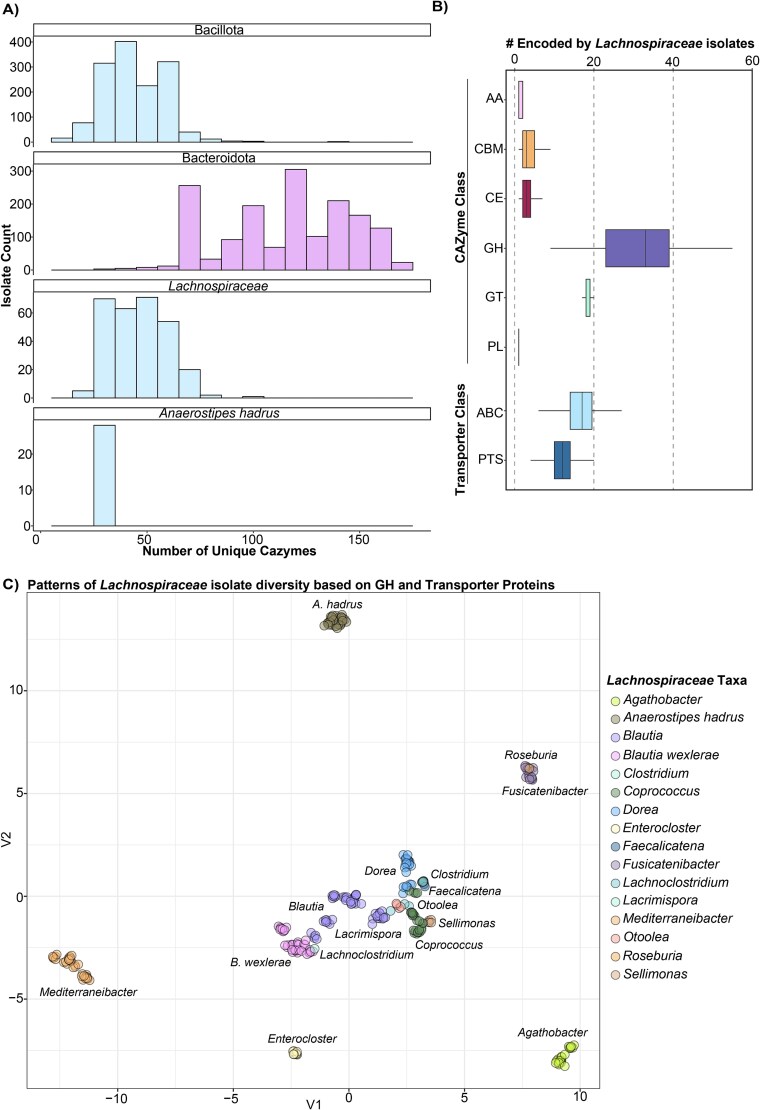
*Lachnospiraceae* encode broad carbohydrate-active enzymes (CAZymes) and carbohydrate transporter repertoires. (A) Number of unique CAZyme families/subfamilies annotated in isolate genomes from members of the *Bacillota* phylum, *Bacteroidota* phylum, *Lachnospiraceae* family, and *Anaerostipes hadrus* species. (B) Number of unique CAZyme families/subfamilies and unique carbohydrate transporter systems from the PTS and ABC protein families across *Lachnospiraceae* isolates. (C) UMAP of *Lachnospiraceae* based on the variable numbers of genes annotated as different carbohydrate transporters by Prokka and or GHs by dbCAN in each isolate. Each point represents an isolate and is colored according to the genus and species identification as indicated.

Given the importance of nutrient niche occupancy, we investigated how genomic diversity in *Lachnospiraceae* alters nutrient niche access. First, we annotated CAZymes across several isolate biobanks [23, 36, 37]. As expected, Bacillota and *Lachnospiraceae* isolates encode fewer unique CAZymes than Bacteroidota isolates, with most *Lachnospiraceae* encoding 25–50 unique CAZymes (Fig. 1A). Including multiple copies, *Lachnospiraceae* isolates encode a median of 54 GH proteins in 33 different GH families, suggesting a substantial ability to act on different carbohydrates (Fig. 1B). We also assessed the repertoire of PTS and ABC transporter families encoded by *Lachnospiraceae*, and found that isolates encode a median of 17 and 12 unique ABC and PTS transporter systems, respectively (Fig. 1B). Next, we investigated patterns of GH and transporter distribution across *Lachnospiraceae*. Based only on differential encoding of these proteins, *Lachnospiraceae* isolates formed distinct clusters corresponding to their species identification (Fig. 1C). This indicates that different species encode a characteristic suite of CAZymes. Interestingly, AH isolates clustered separately from other *Lachnospiraceae* (Fig. 1C). Therefore, we focused our subsequent analyses on AH using 19 isolates isolated from 7 healthy human donors [23] (Table.S2). These AH isolates encode a median of 30 different CAZyme families (Fig. 1A).

**Figure 2:**
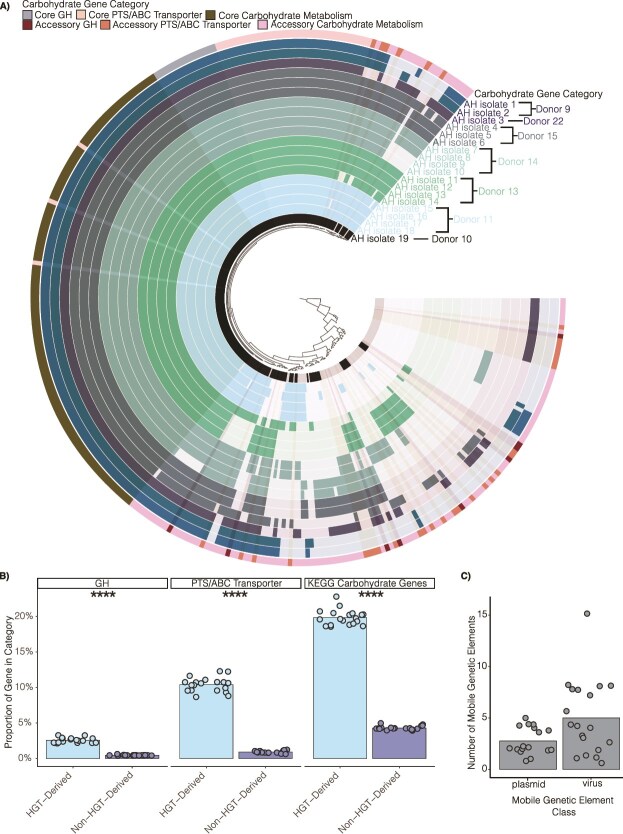
*Anaerostipes hadrus* isolates show extensive strain-level variation in carbohydrate metabolism-related genes. (A) Circular phylogram of 19 AH isolates based on aggregated functional annotation of unique GH families/subfamilies, unique carbohydrate transporter genes, and other unique carbohydrate metabolism genes. The outermost ring represents the carbohydrate gene categories, and all other rings represent AH isolates, which are colored by donor. (B) Gene proportion of carbohydrate genes assigned in predicted categories of HGT-derived and non-HGT-derived genes for GHs, PTS/ABC transporters, and KEGG annotated carbohydrate genes. The proportions were calculated from the total number of coding genes putatively annotated as HGT-derived by HGTector2 and all other genes not assigned to be HGT-derived. A two-tailed t-test was performed and ^****^ indicate a *P* < .0001. (C) Number of MGEs per genome as annotated by geNomad.

Interestingly, the presence of GH protein families, PTS and ABC transporter genes, and carbohydrate metabolism genes varied extensively between individual AH isolates (Figs 2A, S1A). Indeed, approximately equal numbers of these genes are present in the core and accessory genome of these AH isolates (Figs 2A, S1B). Interestingly, in multiple instances, not all AH isolates from the same donor encoded the same set of carbohydrate-related genes (Fig. 2A). This strain-level variation was not reflected in the full-length 16S rRNA identity (99.77% average) or the ANI (98.45% average) (Fig. S1C and D).

To investigate if AH strain variation in carbohydrate-related genes was associated with HGT we used HGTector2 [44]. Across AH isolates, on average 510 genes per isolate were predicted to be HGT-associated (Fig. S1E). Interestingly, for each AH isolate, carbohydrate-related genes represented a greater proportion of the HGT-associated gene subset than the non-HGT associated genes (Fig. 2B). Consistent with extensive HGT, AH isolates encoded multiple MGEs as predicted by geNomad (Figs 2C, S1F) [45]. Therefore, HGT may support differential nutrient niche access between closely related AH strains.

### Strain-level variation in nutrient niche access

**Figure 3:**
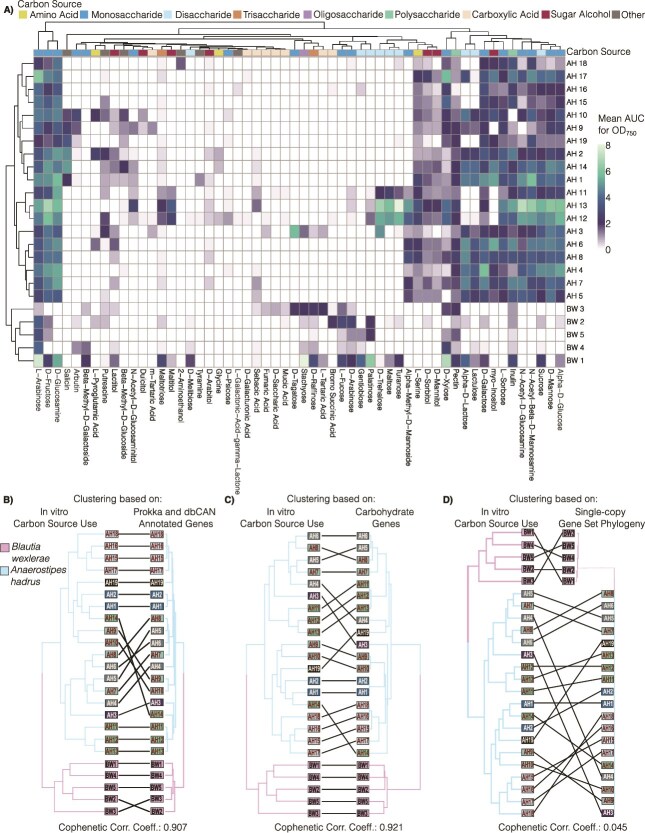
Nutrient niche access of *Anaerostipes hadrus* isolates varies at a strain level. (A) Heatmap depicting 56 carbon sources that at least 1 isolate of 19 AH and 5 BW grew on. Values plotted represent the mean AUC for measured OD_750_ over 24 h across two independent runs. Isolates are clustered based on the carbon source use via hierarchical clustering using Euclidean distance. (B–D) Tanglegrams of the dendrogram cophenetic correlation of 19 AH and 5 BW isolates based on clustering of the *in vitro* carbon source use to the cluster based on annotation of all coding genes from Prokka and CAZymes by dbCAN (B), only carbohydrate genes (C), and a single-copy gene set phylogeny (D). All clustering was performed using the Euclidean distance, and the phylogenomic tree was generated via GtoTree as described in the methods.

To assess nutrient niche access, we measured growth of 19 AH and 5 BW, another member of *Lachnospiraceae*, on a screening panel of 190 carbon sources that includes simple carbohydrates, amino acids, several oligo- or poly-saccharides and other compounds as sole carbon sources (Table S2 and Fig. S2A). Under the tested conditions, 56 different compounds, including 9 disaccharides and 18 monosaccharides, supported growth of at least one isolate (Figs 3A, S2B). In addition, most AH isolates grew on the polysaccharides inulin or pectin, while select strains grew using the tri- or oligosaccharides maltotriose, raffinose, or stachyose (Figs 3A, S2B). For widely used carbon sources, doubling times were similar between strains and carbon sources (Fig. S2C). Overall, AH isolates had a distinct profile from BW isolates, and the use of some carbon sources, such as myo-inositol and L-fucose, was species-restricted (Fig. 3A). Interestingly, this analysis also revealed widespread strain-level differences. For example, AH strains varied in growth on D-trehalose, stachyose, inositol, lactose, and lactulose (Fig. 3A).

Next, we investigated how the observed functional differences correlated with strain-level genomic variation. First, we annotated isolate genomes with Prokka and combined this with CAZyme annotations. Based on this annotation, AH and BW isolates formed two distinct clades (Fig. 3B). Interestingly, carbon use profiles strongly correlated (r0.907) with this clustering of isolates (Fig. 3B). Next, we focused the annotation dataset to only include carbohydrate-related genes, including GHs, PTS and ABC transporters, and other carbohydrate metabolism genes (Fig. S1A). Gene distribution patterns in this focused gene set also strongly correlated (r0.921) with patterns of carbon use (Fig. 3C). In contrast, carbon source use did not correlate (r0.045) with isolate phylogeny based on sequence variation in a single-copy gene set (Fig. 3D).

### Gene cluster gain, loss, and disruption dictate nutrient niche access

**Figure 4:**
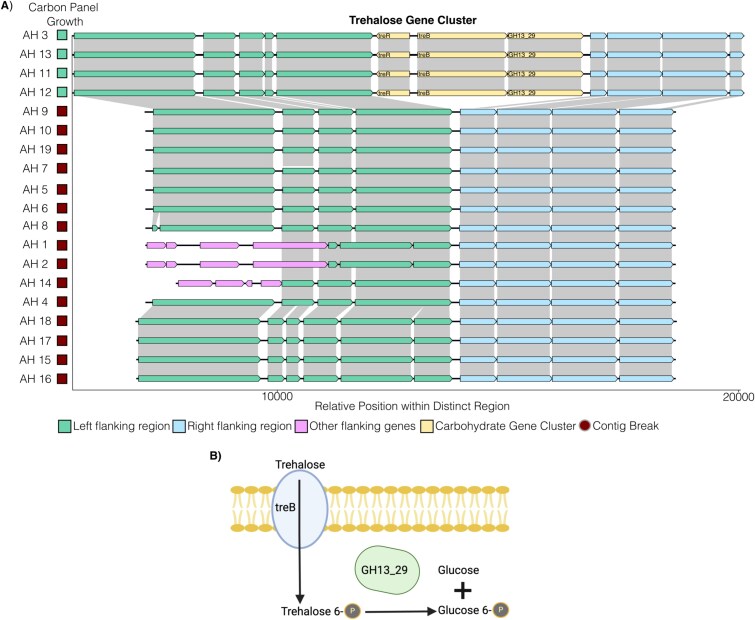
*Anaerostipes hadrus* isolates variably encode a gene cluster for trehalose use. (A) Identification of a predicted trehalose use gene cluster in four trehalose metabolizing AH isolates based on annotation data from Prokka and dbCAN. The identified genomic region for each gene cluster has been highlighted with annotated genes. The ability of each isolate to grow in carbon use screening is indicated. (B) Metabolic schematic of how the identified gene cluster supports trehalose metabolism.

To identify genomic changes that explain variable nutrient niche access, we first examined carbohydrates that were used by a few strains, including trehalose and stachyose. We identified a putative trehalose gene cluster consisting of a regulator, import protein, and GH (GH13_29) that was present exclusively in the four trehalose-utilizing strains (Fig. 4A and B). This gene cluster was in a region that was conserved in nontrehalose utilizing strains (Fig. 4A). Similarly, the only AH isolate capable of stachyose utilization encoded a corresponding gene cluster in a conserved genomic region (Fig. S3A and B). This AH stachyose gene cluster was distinct from a related gene cluster in stachyose-utilizing BW (Fig. S3C).

**Figure 5:**
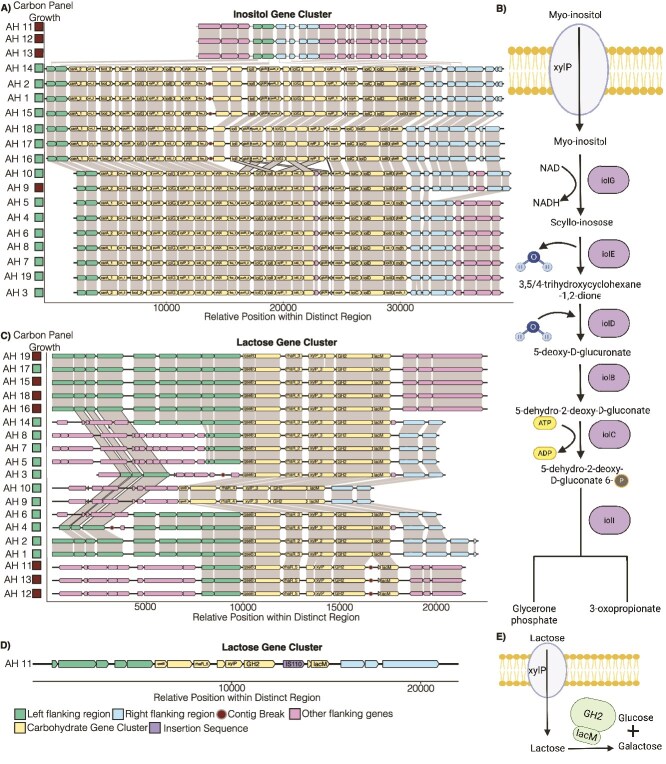
Genomic changes in AH isolates explain variable inositol and lactose metabolism. (A, C) identification of predicted carbohydrate utilization gene clusters in 19 AH isolates based on annotation data from Prokka and dbCAN for inositol (A) and lactose (C). The identified genomic region for each gene cluster has been highlighted with annotated genes. The ability of each isolate to grow in carbon use screening is indicated. (D) Putative carbohydrate utilization gene cluster for lactose based on long-read sequencing for AH 11. (B, E) metabolic schematics of how the identified gene clusters support inositol (B) and lactose (E) metabolism.

Most, but not all, AH strains use lactose and inositol (Fig. 3A). In agreement, we identified a gene cluster encoding multiple inositol utilization genes present in all tested strains apart from three, which did not show growth on inositol (Fig. 5A and B). In these three AH, the cluster-flanking genes were adjacent in the genome, suggesting a potential loss of the gene cluster (Fig. 5A). Unexpectedly, AH9 encoded the inositol gene cluster but did not grow on inositol in the initial carbon source screening (Fig. 5A).

Unexpectedly, we identified a putative lactose gene cluster that was present in all isolates, including AH isolates that did not grow on lactose under the tested conditions (Fig. 5C and E). However, for three of the isolates that were unable to use lactose (AH11/12/13), a truncation was predicted in a transporter gene along with a break in the assembled contig, suggesting that genomic rearrangements and/or mutations had occurred at this site (Fig. 5C). To investigate this further, we used long-read sequencing, generating a circularized genome, and re-examined this region. Interestingly, this revealed the presence of an insertion sequence at this site (Fig. 5D). We noted that AH15/16/18/19 encoded a lactose gene cluster without this insertion sequence, but still did not grow on lactose in the carbon screening panel (Fig. 3A) where single carbon sources are provided in a base inoculating media.

To investigate instances where isolates encoded the corresponding gene cluster but did not grow under the conditions of the carbohydrate panel, we tested growth in an alternative nutrient environment using BHI without dextrose (BHI-D). Without additional supplementation, AH isolate growth was low in BHI-D (Fig. S4). When inositol was added, all inositol gene cluster encoding AH isolates, including AH9, which did not grow using inositol on the screening panel (Fig. 3A), showed enhanced growth (Fig. S4). Interestingly, when lactose was added, the AH15/16/18/19 isolates, which were negative on the screening panel, were able to grow, albeit with a longer lag phase compared to other lactose-utilizing strains (Fig. S4). Overall, this indicates that, for multiple carbohydrates, the ability of AH strains to occupy nutrient niches can be traced to the gain, loss, or disruption of specific gene clusters, but is also influenced by other components of the growth media.

### Variable nutrient niche access drives differential SCFA production

**Figure 6:**
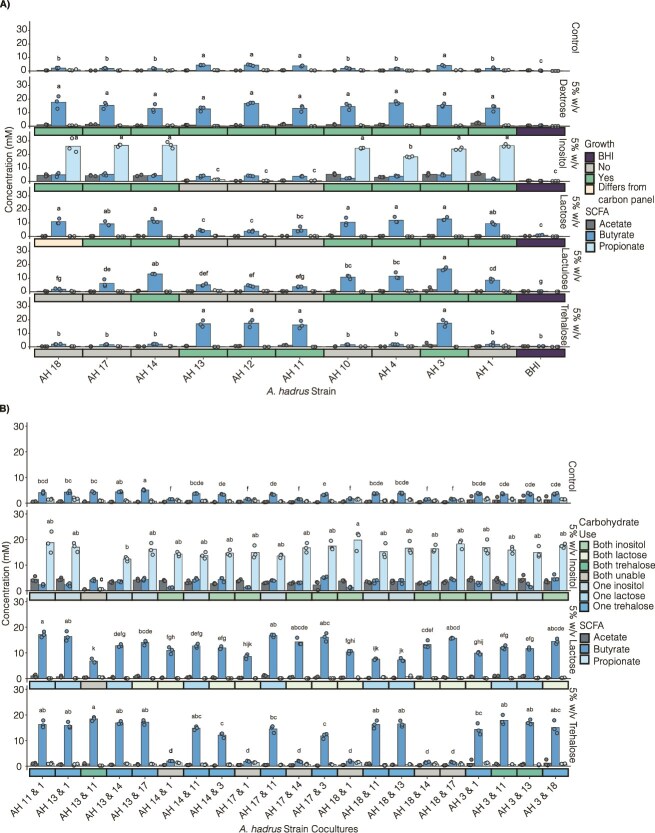
SCFA production by AH strains varies in environments with a single carbohydrate niche. (A) SCFA production in 10 AH isolates grown in BHI without dextrose supplemented with 5% w/v dextrose, inositol, lactose, lactulose, or trehalose. The predicted growth based on the carbon source screening panel is indicated. (B) SCFA production for 21 co-cultures with 7 AH isolates in indicated combinations. The isolates were grown 24 h in BHI without dextrose supplemented with 5% w/v inositol, lactose, or trehalose. The predicted carbohydrate use of the two isolates in each combination is indicated. All analyses were performed with a one-way ANOVA followed by a *post hoc* Tukey test, with letters indicating statistical significance (*P* < .05). Each point represents an independent biological replicate.

Next, we asked how growth on different carbon sources alters SCFA production by AH. We focused on inositol, lactulose, lactose, and trehalose and a subset of AH strains that showed variable growth on these carbohydrates. In BHI-D, SCFA production was low at baseline, but all isolates significantly increased butyrate production with dextrose supplementation, consistent with the universal ability of these AH strains to grow on dextrose (Figs 6A, 3A). Lactose, lactulose, or trehalose supplementation increased butyrate production in a unique, strain-specific pattern that corresponded to isolate growth and gene cluster presence (Fig. 6A). In contrast, inositol supplementation triggered propionate production in a strain-specific manner corresponding to the presence of the identified gene cluster (Fig. 6A), consistent with earlier reports [51]. Next, we asked if co-culturing AH strains altered niche occupancy in carbohydrate-supplemented BHI-D, and found that SCFAs were produced at similar levels if at least one of the strains could access the nutrient niche (Fig. 6B).

Next, we tracked growth and SCFA production over time by AH3, which uses all the tested carbohydrates (Figs 6A, S5). This revealed that the lag phase and growth rate remained consistent between different carbohydrate conditions (Fig. S5A). Furthermore, the levels and kinetics of butyrate production across timepoints was similar between cultures with lactose, lactulose, trehalose and dextrose, while propionate was produced with similar kinetics in inositol cultures (Fig. S5). There were modest, but significant differences in the maximum optical density (OD) reached by AH3 on different sugars, but these did not correspond to changes in SCFA concentrations (Fig. S5).

### Strain-specific nutrient niche preferences

**Figure 7:**
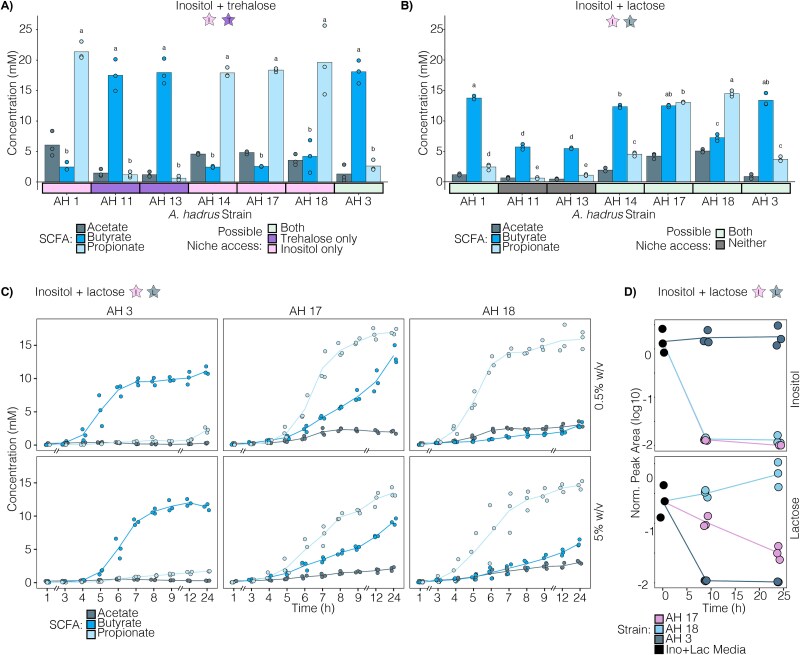
AH in nutrient mixes reveal distinct SCFA profiles and varying consumption of carbon sources. (A, B) SCFA production of seven AH isolates grown in BHI without dextrose supplemented with nutrient mixes of 5% w/v inositol + trehalose (A) and 5% w/v inositol + lactose (B). Possible carbohydrate niche access is indicated below the bar graphs. (C) SCFA production of AH3, AH17, and AH18 grown in 0.5% w/v and 5% w/v inositol + lactose at hours 1, 3–9, 12, and 24 h. (D) Sugar consumption by AH 3, AH 17, and AH18 of inositol + lactose compared to baseline media at hours 9 and 24. Analyses were performed with a one-way ANOVA followed by a *post hoc* Tukey test, with letters indicating statistical significance (*P* < .05). Each point represents an independent biological replicate.

In a competitive environment, a microbe’s realized nutrient niche is expected to be smaller than the range of niches it can potentially occupy [7]. This can be influenced by several factors including competition with other microbes or preferential substrate use. To examine preferential carbohydrate use, we compared SCFA production by AH isolates on mixtures of inositol+trehalose or inositol+lactose. Here, inositol use will produce propionate, while trehalose or lactose use will yield butyrate. As expected, when a strain can only access one nutrient niche, the corresponding SCFA is produced (Fig. 7A and B). AH3, which is the only strain able to use either inositol or trehalose (Fig. 6A), predominantly produced butyrate in inositol+trehalose conditions indicating preferential trehalose use (Fig. 7A). Interestingly, five of the tested AH strains can use either inositol or lactose (Fig. 6A). Strikingly, these isolates showed distinct preferences in inositol+lactose conditions (Fig. 7B). AH1, AH3, and AH14 produced butyrate and limited propionate while AH17 produced both propionate and butyrate and AH18 predominantly produced propionate (Fig. 7B). To further investigate these preferences, we measured SCFA production over time on inositol+lactose (Fig. 7C). As expected, AH3 produced butyrate and AH18 produced propionate throughout the time course (Fig. 7C). Interestingly, AH17 produced propionate and butyrate with similar kinetics, consistent with simultaneous metabolism of both carbohydrates. These strain-specific patterns of SCFA production were maintained at 10-fold lower levels of carbohydrate supplementation, which facilitated analysis of carbohydrate consumption (Fig. 7C). We observed that AH3 rapidly depleted lactose but not inositol, AH18 depleted inositol but not lactose, and AH17 depleted both sugars, consistent with the observed SCFA production patterns (Fig. 7D).

### Intra-species interactions influence AH nutrient niche access

**Figure 8:**
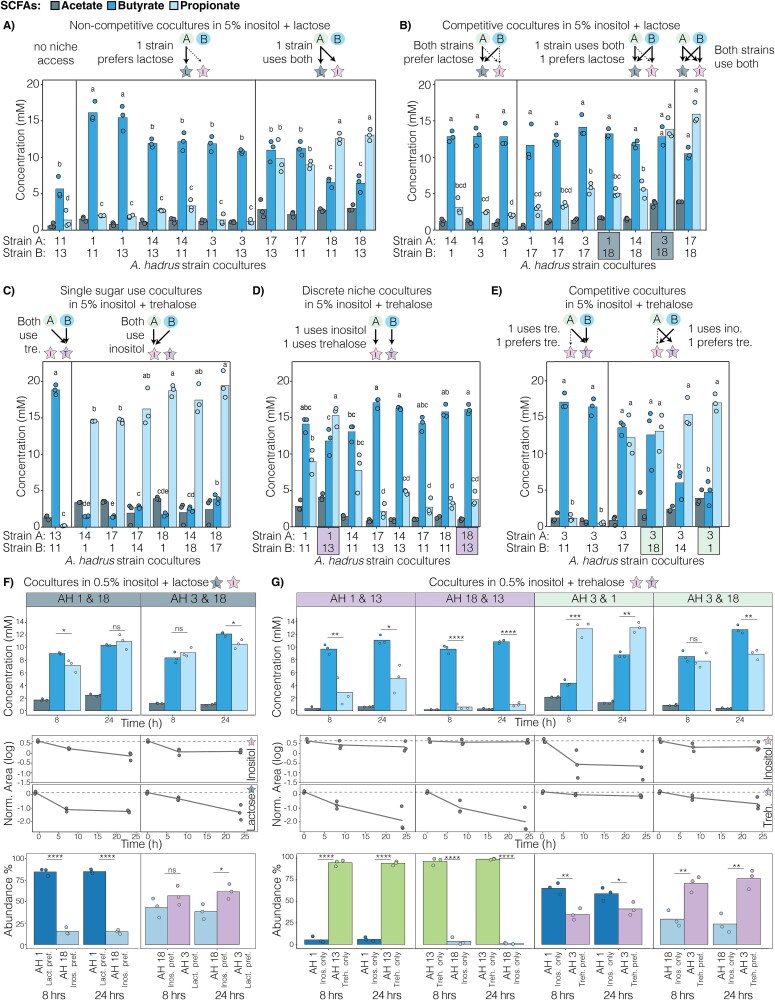
AH co-cultures show unique SCFA profiles that vary based on nutrient mix, differences in sugar consumption, and strain interactions. (A, B) AH co-cultures grown in a nutrient mix of 5% w/v inositol + lactose. (A) SCFA production for 11 noncompetitive AH co-cultures grouped by no niche access, one strain preferring lactose, and one strain using both. (B) SCFA production for 10 competitive AH co-cultures grouped by both strains preferring lactose, one strain using both and one preferring lactose, and both strains using both carbohydrates. (C, D, E) AH co-cultures grown in a nutrient mix of 5% w/v inositol + trehalose. (C) SCFA production for seven AH co-cultures where both strains use a single carbohydrate, grouped by a co-culture of both using trehalose and co-cultures of strains using inositol. (D) SCFA production for eight AH co-cultures where one isolate uses inositol and one uses trehalose. (E) SCFA production for six competitive AH co-cultures grouped by co-cultures of one strain using trehalose and one preferring trehalose and co-cultures of one using inositol and one preferring trehalose. All analyses were performed with a one-way ANOVA followed by a *post hoc* Tukey test. (F) Two AH co-cultures grown in 0.5% w/v inositol + lactose with measured SCFA production, sugar consumption of inositol and lactose, and percent abundance of both co-cultured AH at 8 and 24 h. (G) Four AH co-cultures grown in 0.5% w/v inositol + trehalose with measured SCFA production, sugar consumption of inositol and trehalose, and percent abundance of both co-cultured AH at 8 and 24 h. Two-tailed t-test was performed for comparisons of butyrate and propionate production and for percent abundance of co-cultures. The ^*^, ^**^, ^***^, and ^****^ indicate a *P*-value <.05, <.01, <.001, and < .0001, respectively and each point represents an independent biological replicate.

Next, we investigated SCFA production in AH strain co-cultures with two carbohydrate niches. First, strains were co-cultured with inositol+lactose (Fig. 8A and B). In conditions where one strain was unable to use either carbohydrate while the other could use both, the SCFA profile reflected the carbon source preferences of the single utilizing isolate (Fig. 7A), resulting in a range of butyrate:propionate ratios (Fig. 8A). Interestingly, when both AH strains could use either inositol or lactose, co-cultures showed varied SCFA profiles (Fig. 8B). When two strains that preferentially use lactose (AH1, 14, or 3—Fig. 7A) were co-cultured this did not alter carbohydrate preference, and the SCFA profile reflected lactose use with butyrate predominating (Fig. 8B). When strains with preference for inositol (AH17 or 18—Fig. 7A) were co-cultured with lactose-preferring strains, the SCFA profile of the co-culture often reflected lactose use (Fig. 8B). However, co-culture of AH3 (lactose preference) and AH18 (inositol preference) led to both SCFAs being produced. Finally, co-culture of two strains with higher inositol preference (AH17 and 18) supported both butyrate and propionate production (Fig. 8B).

Next, we examined co-cultures in inositol+trehalose. As expected, conditions where both isolates competed either for inositol or trehalose triggered only propionate or butyrate production, respectively (Fig. 8C). Interestingly, in inositol+trehalose co-cultures where one isolate only uses trehalose and the other only uses inositol, despite the lack of competition for these sugars, we observed several different SCFA profiles (Fig. 8D). In some co-cultures, SCFA production reflected metabolic activity of both strains (butyrate+propionate production), while in others only butyrate was detected (Fig. 8D). Finally, we examined a set of co-cultures that included AH3, which can use both inositol and trehalose but preferentially metabolizes trehalose in an inositol+trehalose mixture (Figs 7A, 8E). When co-cultured with strains only able to use trehalose (AH11 and 13) only butyrate was produced. Interestingly, when AH3 was co-cultured with four different strains that were only able to use inositol, we observed a range of SCFA profiles with varying levels of butyrate and propionate production (Fig. 8E).

Given these observations of complex interactions, we selected conditions for additional analysis by choosing three pairs of co-cultures that share an AH strain and unexpectedly differ in SCFA production despite the constituent strains having the same carbohydrate use capabilities or preferences in monocultures (Fig. 8B, D and E—highlighted). We used 0.5% w/v of carbohydrates to facilitate analysis of carbohydrate consumption, and found that, after 8 h, the relative differences in butyrate or propionate production between selected pairs of isolates remain consistent with patterns at higher carbohydrate levels (Fig. 8B, D–G). However, particularly by 24 h, several of these co-cultures yielded high levels of both propionate+butyrate in contrast to single SCFA production (propionate or butyrate) observed when carbohydrates were supplied at 5% w/v (Fig. 8F,G Top). As anticipated, increased butyrate production occurred alongside greater consumption of lactose or trehalose, while increased propionate production corresponded to increased inositol consumption (Fig. 8F,G Middle).

Next, we measured the abundance of individual AH strains in these co-cultures. This revealed that SCFA production and carbohydrate use patterns reflect varying abundances of the two strains and the behavior of those strains in monocultures (Fig. 8F and G). For example, in inositol+trehalose, when AH3 (trehalose preference) is co-cultured with AH1 (inositol use), AH1 significantly outcompetes AH3, leading to propionate production and inositol consumption (Fig. 8G). In contrast, when AH3 is grown instead with AH18 (inositol use) in inositol+trehalose, AH3 significantly outcompetes AH18, leading to relatively higher levels of butyrate production and trehalose consumption (Fig. 8G). In a second pair of conditions, co-culture of AH13 (trehalose use) with AH1 (inositol use) in inositol+trehalose leads to more propionate production than when AH13 is grown with AH18 (inositol use) (Fig. 8G). In line with this, after 24 h, AH1 is more abundant than AH18 when in co-culture with AH13 (Fig. 8G). Consistent with the overall dominance of AH13 (trehalose use), high levels of butyrate are produced in both co-cultures, and trehalose is consumed (Fig. 8G). In a third example, after 8 h in inositol+lactose, AH18 (inositol preference) competes more successfully with AH3 (lactose preference) than AH1 (lactose preference), leading to relatively higher levels of propionate compared to butyrate and less lactose consumption at this timepoint (Fig. 8F). By 24 h, AH18 + AH3 co-cultures deplete more of the lactose and increase butyrate production relative to propionate, while AH18 + AH1 co-cultures use more of the inositol and increase propionate relative to butyrate (Fig. 8F).

## Discussion

Competition for nutrient niches and the production of fermentation metabolites, including SCFAs, is critical for the assembly and host-benefitting functions of the microbiota [52, 53]. In this study, we found that AH strains vary in their nutrient niche access, have distinct preferences for different carbohydrates, and show complex strain–strain interactions in co-cultures. Interestingly, our analyses indicate that individuals can be colonized by multiple AH strains that vary in niche access and carbohydrate preferences.

We found that AH strains use multiple mono- and disaccharides. In this screening, the polysaccharides pectin and inulin also supported AH growth (Fig. 3A). However, the screening panel only contained 190 different carbon sources and includes a limited number of polysaccharides (Figs 3, S2). Therefore, expanding screening to include a wider range of dietary polysaccharides could provide additional insight into the nutrient niche occupancy of AH. In some members of Bacillota, including isolates of *Roseburia*, the presence of Gram-positive PULs (gpPULs) has been described [20, 54]. It will be interesting to test if gpPULs in AH or other *Lachnospiraceae* show similar strain variation as simple sugar gene clusters in AH.

HGT distributes antibiotic resistance genes in the microbiota, and much of our understanding of HGT has come from studies on antibiotic resistance [55–57]. However, recently developed bioinformatics pipelines have indicated that, within the gut microbiota, Bacteroidota and Bacillota also transfer carbohydrate utilization genes [58, 59]. In agreement, our analyses indicate that the analyzed AH strains have multiple predicted MGEs and have acquired some of their carbohydrate-related genes through HGT (Figs 2, 4, 5). Indeed, an isolate’s carbon source use profile correlates with varying presence/absence of carbohydrate-related genes (Fig. 3C). Therefore, the specific HGT mechanisms through which AH gain and transfer carbohydrate use gene clusters within a community warrants additional investigation.

We identified gene clusters for use of carbohydrates, including trehalose, stachyose, lactose, and inositol, which explain variable access to these nutrient niches (Figs 4, 5, S3). These clusters can occur in otherwise conserved genomic regions. While carbohydrate gene cluster distribution explains most patterns of strain-restricted niche access, we also noted instances where isolates encoded gene clusters for inositol (AH9) or lactose (AH15,16,18,19) but did not grow in our initial screening panel. Interestingly, in these cases, all isolates showed growth in an alternate nutrient environment—BHI-D with inositol or lactose—either at the same rate as other strains (AH9 on inositol) or with a longer lag phase (AH15,16,18,19 on lactose) (Fig. S4). We also anticipated that, given a growth environment with two carbohydrate sources, isolates with the same underlying abilities to use the provided carbohydrates would show the same carbohydrate preferences. Unexpectedly, we observed that SCFA production and carbohydrate preference in mixed carbohydrate environments varies by strain (Fig. 7). For example, in inositol+lactose conditions, AH3, AH17, and AH18 vary in inositol or lactose consumption leading to different levels of propionate verse butyrate produced (Fig. 7) even though all three produce similar levels of butyrate or propionate when grown on lactose or inositol alone (Fig. 6). Interestingly, AH17 uses both lactose and inositol with similar kinetics (Fig. 7C). Together, these observations of strain-specific carbohydrate preferences or varying abilities to use carbohydrates in different environments indicate that factors beyond the presence of the respective gene cluster may influence carbohydrate use. These factors could include the regulation of gene cluster expression, requirements for other media components when using a given carbohydrate, and the influence of genes beyond the identified conserved gene clusters. Indeed, these AH strains have an extensive accessory genome [23]. Collectively, these additional factors could influence how advantageous the use of different carbohydrates is in a specific strain context and await further investigation. For example, the use of transcriptomics to identify changes in gene expression outside of identified gene clusters or differential gene cluster regulation could be informative. These findings also have implications for live biotherapeutic development. Given the complex nutrient environment of the gut, these results highlight the importance of empirical investigation of specific strain behavior in a target environment.

The AH gene cluster corresponding to inositol use includes both genes with known functions in inositol metabolism and other proteins, including unannotated/hypothetical proteins, with uncharacterized roles in the metabolic pathway (Fig. 5A). Similarly, putative stachyose-use clusters differed between AH and BW strains and contained un-annotated/hypothetical proteins (Fig. S3). Therefore, additional work will be needed to characterize the detailed biochemical mechanism through which AH can metabolize the tested carbohydrates, and how these metabolic capabilities are regulated and integrated into the overall metabolic network of individual strains.

Previous studies have examined co-cultures of other Gram-positive gut microbiota members [60, 61] using combinations of different species or strains of *Lactobacillus* [61–63]. Our work extends these findings to the strain level in AH and reveals that the complexity of individual strain behavior with multiple possible nutrient niches is further amplified when multiple strains are co-cultured. Indeed, our data indicate that there are strain–strain interactions that influence the relative abundance of two strains in co-cultures, which alter the resulting SCFA profiles and carbohydrate consumption. Therefore, interactions between strains and results of co-cultures are not always predicted by the additive combination of individual strain behaviors in the same environments (Fig. 8). The pathways and functions that mediate these strain–strain interactions beyond the ability to access available nutrient niches remain to be elucidated. Behavior of co-cultures can also be influenced by the abundance of carbohydrates in the environment. For example, when carbohydrates are provided at 0.5% w/v, initially lactose is rapidly depleted by AH1 + 18 co-cultures followed by a switch to inositol consumption. In contrast, at 5% w/v, where sugars are not limiting, SCFA production reflects lactose use without a significant switch to inositol use. Given that competition for nutrients and SCFA production are recognized as important parameters of colonization resistance [13, 64], these results indicate that a rationale design approach for live biotherapeutic products will be aided by or require consideration of isolate inclusion at a strain level [35].

Overall, this study demonstrates that AH strains vary in their ability to access different carbohydrate nutrient niches, resulting in strain-specific SCFA production. These capabilities are associated with the presence, absence, or disruption of gene clusters enabling use of specific carbon sources. Furthermore, AH strains that are all capable of metabolizing different carbohydrates in isolation show variable preferences for those carbohydrates in mixed nutrient environments. This strain-specific behavior also results in complex interactions between AH strains in co-culture, as reflected in varying SCFA profiles and carbohydrate consumption. Altogether, this highlights the importance of considering the activities and interactions of specific strains in the context of live-biotherapeutic development.

## Supplementary Material

Supplementary_Figure_Captions_ycaf163

Table_S1_ycaf163

Table_S2_ycaf163

Table_S3_ycaf163

Table_S4_ycaf163

## Data Availability

All data generated or analyzed during this study are included in this article (and its supplementary information files) or are available on NCBI (Strain details and raw data in Table S1 and S4). AH and BW strains are available through the Symbiotic Bacterial Strain Bank at the University of Chicago.
