# Supplementary material for: Strain-level variation controls nutrient niche occupancy by health-associated Anaerostipes hadrus
Source: ISME Commun. 2025 Sep 17;5(1):ycaf163. doi: 10.1093/ismeco/ycaf163 (PMC12503163; doi:10.1093/ismeco/ycaf163)
Supplement: Supplementary_Figure_Captions_ycaf163 [file supplementary_figure_captions_ycaf163.pdf]

Supplementary Fig. 1 | *Anaerostipes hadrus* isolates exhibit a varied accessory genome for carbohydrate metabolism A) Methodological overview showing the annotation gene set used for strain analyses that combines GH annotation from dbCAN, transporter annotation by Prokka, and other carbohydrate metabolism gene annotation via KEGG. B) Number of unique core and accessory GH families/subfamilies, unique PTS and ABC transporter genes, and other unique carbohydrate metabolism genes in 19 *Anaerostipes hadrus* (AH). C, D) Matrices of the 16S similarity (C) and average nucleotide identity score (D) for the pairwise comparison of the 19 AH isolates. E) Total number of coding genes present in all 19 AH isolates. The coding genes were putatively classified as HGT-derived or non-HGT-derived via HGTector2. F) Table of the number of mobile genetic elements for each of the 19 AH isolates as designated by geNomad.

Supplementary Fig. 2 | Number and types of carbon sources on screening panel show no differences in doubling time A) Number and types of the 190 different carbon sources present on the carbon source screening panel. B) Number and types of the 56 different carbon sources that showed growth by at least one isolate on the carbon source screening panel. C) Doubling time of *A. hadrus* and *B. wexlerae* isolates that showed growth on 10 different carbon sources and each point is the mean across 2 independent runs.

Supplementary Fig. 3 | Identified strain-level dependent carbohydrate gene cluster for stachyose in *Anaerostipes hadrus* and *Blautia wexlerae*. A) Identification of a predicted carbohydrate utilization gene cluster for stachyose across 19 *A. hadrus* isolates based on annotation data from Prokka and dbCAN. The identified genomic region for the gene cluster is highlighted in yellow. B) Metabolic schematic of how the identified gene cluster supports stachyose metabolism. C) Identification of a predicted carbohydrate utilization gene cluster for stachyose in 4 *B. wexlerae* that grew on stachyose in the carbon source panel screening.

Supplementary Fig. 4 | *Anaerostipes hadrus* isolates demonstrate growth on carbon sources in supplemented BHI w/o dextrose media. Growth curves of *A. hadrus* isolates grown in BHI without dextrose alone and supplemented with 5% w/v lactose or 5% w/v inositol. Isolate growth was measured for 24 hours at OD_{600} across 3 independent runs.

Supplementary Fig. 5 | *Anaerostipes hadrus* isolate 3 reveals similar butyrate production across carbon sources. A) Growth of *A. hadrus* isolate 3 in BHI without dextrose alone and supplemented with 5% w/v dextrose, inositol, lactose, lactulose, or trehalose. Isolate growth was measured at hours 1, 3–9, 12, and 24 hours at $OD_{600.}$ B-D) Butyrate, acetate, and propionate production of AH3 grown in BHI without dextrose alone and supplemented with 5% w/v dextrose,

inositol, lactose, lactulose, or trehalose. SCFA production was quantified at hours 1, 3–9, 12, and 24 hours. A one-way ANOVA was performed at timepoints 5–9, 12, and 24 hours for sugar comparison and each point represents an independent biological replicate.
