# Supplementary material for: Strain-level variation controls nutrient niche occupancy by health-associated Anaerostipes hadrus
Source: ISME Commun. 2025 Sep 17;5(1):ycaf163. doi: 10.1093/ismeco/ycaf163 (PMC12503163; doi:10.1093/ismeco/ycaf163)
Supplement: Table_S1_ycaf163 [file table_s1_ycaf163.docx]

**Table S1. Bacterial Isolates**

| Species | Isolate | NCBI Strain ID | NCBI RefSeq # | Reference | Donor |
| --- | --- | --- | --- | --- | --- |
| Anaerostipes hadrus | AH 1 | MSK.9.9 | GCF_013302355.1 | 23 | 9 |
| Anaerostipes hadrus | AH 2 | MSK.9.11 | GCF_013302445.1 | 23 | 9 |
| Anaerostipes hadrus | AH 3 | MSK.22.35 | GCF_013302475.1 | 23 | 22 |
| Anaerostipes hadrus | AH 4 | MSK.15.40 | GCF_013302525.1 | 23 | 15 |
| Anaerostipes hadrus | AH 5 | MSK.15.37 | GCF_013302455.1 | 23 | 15 |
| Anaerostipes hadrus | AH 6 | MSK.15.34 | GCF_013303225.1 | 23 | 15 |
| Anaerostipes hadrus | AH 7 | MSK.14.57 | GCF_024460575.1 | 23 | 14 |
| Anaerostipes hadrus | AH 8 | MSK.14.29 | GCF_013302565.1 | 23 | 14 |
| Anaerostipes hadrus | AH 9 | MSK.14.23 | GCF_013302595.1 | 23 | 14 |
| Anaerostipes hadrus | AH 10 | MSK.14.17 | GCF_013302605.1 | 23 | 14 |
| Anaerostipes hadrus | AH 11 | MSK.13.6  ID: 1265725 | GCF_013302645.1  Bioproject # [PRJNA1265725](https://www.ncbi.nlm.nih.gov/bioproject/PRJNA1265725) | 23  re-sequence with long-reads | 13 |
| Anaerostipes hadrus | AH 12 | MSK.13.43 | GCF_013302665.1 | 23 | 13 |
| Anaerostipes hadrus | AH 13 | MSK.13.2 | GCF_013302685.1 | 23 | 13 |
| Anaerostipes hadrus | AH 14 | MSK.13.17 | GCF_013302675.1 | 23 | 13 |
| Anaerostipes hadrus | AH 15 | MSK.11.47 | GCF_013302715.1 | 23 | 11 |
| Anaerostipes hadrus | AH 16 | MSK.11.27 | GCF_013302755.1 | 23 | 11 |
| Anaerostipes hadrus | AH 17 | MSK.11.20 | GCF_013302795.1 | 23 | 11 |
| Anaerostipes hadrus | AH 18 | MSK.11.19 | GCF_013302815.1 | 23 | 11 |
| Anaerostipes hadrus | AH 19 | MSK.10.7 | GCF_013302845.1 | 23 | 10 |
| Blautia wexlerae | BW 1 | MSK.22.49 | GCF_013301765.1 | 23 | 22 |
| Blautia wexlerae | BW 2 | MSK.21.50 | GCF_013304405.1 | 23 | 21 |
| Blautia wexlerae | BW 3 | MSK.15.15 | GCF_013302095.1 | 23 | 15 |
| Blautia wexlerae | BW 4 | MSK.18.42 | GCF_013301935.1 | 23 | 18 |
| Blautia wexlerae | BW 5 | MSK.15.27 | GCF_013302085.1 | 23 | 15 |
